# Incidence of SARS-CoV-2 Co-infections During the Second Wave in Sub-Himalayan Region, India

**DOI:** 10.7759/cureus.36215

**Published:** 2023-03-16

**Authors:** Shailesh Gupta, Ashish Negi, Shailender Negi, Diksha Diksha, Diksha Kandwal, Arpana Singh, Anshu Singh, Pratima Gupta, Deepjyoti Kalita

**Affiliations:** 1 Microbiology, All India Institute of Medical Sciences, Rishikesh, Rishikesh, IND; 2 Microbiology, All India Institute of Medical Sciences, Guwahati, Guwahati, IND

**Keywords:** india, biofire filmarray, sars-cov-2, co-infections, covid-19

## Abstract

Introduction

The second wave of the coronavirus disease 2019 (COVID-19) pandemic in India, which started from April 2021, has been more severe and deadly than the first wave. The aim of this prospective study was to determine the possibility of other respiratory pathogens contributing towards the severity and hospitalization in the current second wave.

Materials and methods

Nasopharyngeal and oropharyngeal swab samples were collected and processed for severe acute respiratory syndrome coronavirus 2 (SARS-CoV-2) by reverse transcription polymerase chain reaction (RT-PCR). These samples were further processed for detection of co-infection in SARS CoV-2 patients by BioFire® Filmarray® 2.0 (bioMérieux, USA).

Results

We screened 77 COVID-19-positive patients admitted to All India Institute of Medical Sciences (AIIMS), Rishikesh and found cases of co-infections in five (6.49 %) patients.

Conclusion

Our finding suggests that co-infections had no or minimal role in augmenting the second wave of the COVID-19 pandemic in India, and the emergence of new variants may be the probable cause.

## Introduction

Coronavirus disease 2019 (COVID-19) is caused by severe acute respiratory syndrome coronavirus 2 (SARS-CoV-2) and was declared a pandemic by World Health Organization (WHO) in March 2020 and within a span of over a year it has affected 169.5 million people and caused death of 3.5 million people across the globe [1]. Patients with COVID-19 typically present with fever, cough, dyspnea, fatigue, anosmia, ageusia, headache, excessive sputum production, and diarrhea. Around 15% of the patients develop severe form of the disease leading to severe pulmonary failure and require hospitalization [2,3].

The prevalence of COVID-19 in India till May 2021 was 28 million and approximately 0.38 million people have lost their lives [4]. The incidence of the cases and deaths have been higher in the second wave of the COVID-19 which started in March 2021 and ended in June 2021. Also, the severity of disease and hospitalization have increased during the second wave. One of the reasons behind this increase is attributed to the emergence of new Delta variant (B.1.617.2) of SARS-CoV-2. Although the new variants might be the cause of increasing number of new cases, co-infections with other respiratory pathogens could probably be a cause for increased number of hospitalization and deaths [5]. Co-infections or secondary infections in COVID-19 positive patients have been reported earlier in the literature. Previous studies mainly focused on healthcare associated infections (HAI) and hospital acquired secondary bacterial infections. Analysis of these studies suggests presence of co-infection or secondary infections in 3-5% of the total COVID-19 positive cases.

Previous studies were mainly focused on HAI, that a patient acquires during the course of treatment. In this study, our goal was to check the possibilities co-infections by other respiratory pathogens, apart from HAI, in the increased incidence of hospitalization and mortality as the second wave of COVID-19 pandemic overlaps with the flu season in India.

## Materials and methods

In this study, samples were taken from the hospitalized patients admitted to the tertiary care setup of All India Institute of Medical Sciences (AIIMS), Rishikesh during the second wave of COVID-19 infection. The samples were collected during the second wave for a period of two months i.e from April 2021 to May 2021. The nasopharyngeal and oropharyngeal swabs were collected during the early stages of hospitalization (within 48 hours) and were first confirmed for SARS-CoV-2 by RT-PCR.

Firstly, we isolated RNA by automatic method using MagMAX Viral/Pathogen Nucleic Acid Isolation Kit (Thermo Fisher Scientific, USA) and KingFisher Duo Prime system (Thermo Fisher Scientific, USA). Then we used TaqPath (Applied Biosystems, USA) COVID-19 RT-PCR Kit to perform RT-PCR in BIO-RAD CFX96 system (Bio-Rad Laboratories, Inc., USA). Only those samples with cT value less than 30 were considered. Co-infections in these positive samples were studied in BioFire® Filmarray® 2.0 system (bioMérieux, USA) using the Respiratory (RP) Plus panel (bioMérieux, USA). The BioFire® Filmarray® 2.0 is a closed system and it employs nested multiplex PCR with a microarray based detection. The turnaround time of the Biofire system is 45 minutes and it can detect 18 respiratory pathogens including 15 viruses; Adenovirus, Coronavirus (HCoV-HKU1, HCoV-NL63, HCoV-OC43), Human Metapneumovirus (HMPV), Human Rhinovirus/Enterovirus (HRV), Influenza viruses (FluA and FluB), Middle East Respiratory Syndrome Coronavirus (MERS-CoV), Parainfluenza types (PIV 1, 2, 3, & 4) and Respiratory Syncytial Virus (RSV), and four atypical bacteria; *Bordetella parapertusis*, *Bordetella pertussis*, *Clamydia pneumonia,* and *Mycoplasma pneumoniae*. The tests were carried as per the manufacturer’s instructions.

## Results

**Figure 1:**
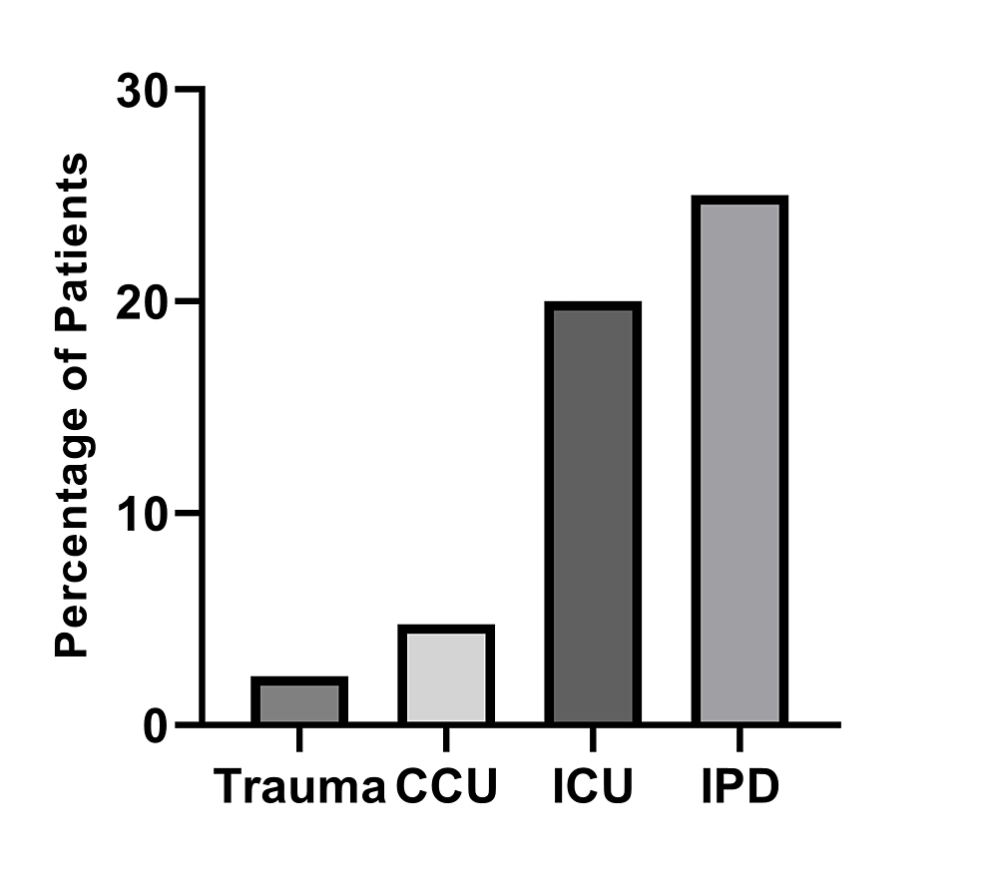
Percentage of patients having co-infections Co-infection percentage of patients admitted to various department were calculated. IPD (25%), ICU (20%), CCU (4.7%), and trauma ED (2.32%). IPD: Invasive pneumococcal disease; ICU: Intensice care unit; ED: Emergency department.

A total of 77 adult COVID-19 positive patients were tested for co-/secondary respiratory pathogens. There were 43 patients from trauma emergency department (ED), 21 from cardiac care unit (CCU), five from intensive care unit (ICU), and eight from in-patient. Out of the 77 patients, there were 53 male patients and 24 female patients with the mean age of 51.75 and 50.12 years, respectively. The age of the patients ranges from 20-94 years. A total of five (6.49%) cases of co-infection were found, out which three cases of other viruses were reported; two Adenovirus (2.59%), one Coronavirus-OC43 (1.29%), and two cases of atypical bacterial infection were reported; *Bordetella parapertusis* (2.59%). All the patients with co-/secondary infections were above the age of 55 years. There was one patient each from trauma ED, CCU, ICU, and two patients from the in-patient department. The percentage of co-infections was higher in patients admitted to ICU (20%) or invasive pneumococcal disease (IPD) (25%) compared to patients admitted to trauma ED (2.32%) or CCU (4.7%) (Figure 1).

## Discussion

This is a randomized prospective study where we found approximately 6% of the SARS-CoV-2 positive patients display co-infection with other respiratory pathogen at the time of testing. Our study is in line with earlier studies (Table 1) which had the same percentage of co-infections.

**Table 1:** Previous studies on co-or secondary infections in COVID-19 patients.

Study	Country	No. of COVID-19 cases	Total of co-infections(%)	Organisms identified (No. of Patients) Viral Bacterial Fungal		
Richarson et al. [6]	USA	5700	42(2.1%)	Rhinovirus/Enterovirus (22), other Coronaviridae (7), RSV(4), Parainfluenza 3 (3), Metapnemovirus(2), and Influenza A (1)	Chamydophila pneumoniae(2) and M. pneumonia(1)	0
Khurana et al. [7]	India	1179	151 (12.8 %)	0	Klebsiella pneumonia, Acinetobacter baumannii, E. coli, and Psedomonas aeruginosa	
Le Hingrat et al. [8]	France	806	55 (6.82 %)	Rhinovirus (17), Coronavirus (15), Adenovirus (7), Parainfluenza (5), Metapnuemovirus (4), Influenza (4) and RSV (2)	6	
Wu et al. [9]	China	201(ARDS)	1(0.6%)	Influenza A (1)	0	0
Zhou et al. [3]	China	191	27(50%)	27 of 54 non-survivors with secondary infections		
Kim et al. [10]	USA	116	24(20.7%)	Rhinovirus/Enterovirus (8), RSV(6), other Coronaviridae (5), Parainfluenza(3), Metapnemovirus(2), and Influenza A (1)	0	0
Ding et al. [11]	China	115	5(4.3%)	Influenza A (3) and Influenza B (2)	0	0
Wang et al. [12]	China	104	6 (5.8%)	Coronavirus (3), Rhinovirus (1), and Influenza A (2)		
Chen et al. [13]	China	99	5 (5.1%)	0	1	Candida albicans (3) and C. glabrata (1)
Zangrillo et al. [14]	Italy	73 (ARDS)		0	Bacterial pneumonia (9, 17.2 %) and Secondary bacteremia (27, 37%)	0
Xing et al. [15]	China	68	25 (36.8%)	Influenza A (18), Influenza B (16), and RSV (1)	M. pneumoniae (8) and Legionella pneumophillia (6)	
Huang et al. [16]	China	41	4(9.8%)	Not Mentioned		
Li et al. [17]	China	40 (Children)	18 (45%)	Influenza A or B (3), Adenovirus (1)	M. pneumoniae (13), Sterptococcus pneumoniae (1)	0
Chen et al. [18]	China	29	1 (3.4%)	NA		
Arentz et al. [19]	USA	21	4 (19%)	3	1	0
Dong et al. [20]	China	11	1 (9%)		Mixture Seen (1)	
Yu et al. [21]	China	7	1 (14%)		Legionella pneumophillia (1)	

The number is proportional to the previous studies [6,8,11-13] and comparable to the first wave of the COVID-19 infection in India [7]. In our study we found two cases each of Adenovirus and *Bordetella parapertusis* and one case of Coronavirus-OC43. While Adenovirus was reported in two of the earlier studies [8,17], there were no reports on *Bordetella parapertusis* as a secondary respiratory pathogen. Though we had a higher percentage of co-infected individuals admitted to ICU or IPD, the patients had similar disease nvasive pneumococcal disease (IPD) of the disease comparable to SARS-CoV-2 infection alone.

The flu season in India peaks around January to March and lasts till May and the second wave in India started in early April and the wave was more severe both in terms of number of infections and hospitalization. Therefore, it was important to assess the contribution of other respiratory pathogens especially influenza to the severity of the second COVID-19 wave. Our findings suggest that the contribution of other respiratory pathogens to the second wave of COVID-19 infection in India may be negligible, and also co-infections have little impact on the disease severity. Hence, the probable cause of the COVID-19 second wave may be the emergence of new variants and further research is required in this direction.

## Conclusions

This is a prospective study where we found approximately 6% of the SARS-CoV-2 positive patients display co-infection with other respiratory pathogen at the time of testing. Our findings of low incidence of respiratory co-infections at the time of hospitalization reinforce that the rising incidence of COVID-19 cases was likely due to the emergence of SARS-COV-2 variants, as reported by other researchers worldwide.
